# Videogame Based Neglect Rehabilitation: A Role for Spatial Remapping and Multisensory Integration?

**DOI:** 10.3389/fnhum.2013.00116

**Published:** 2013-04-02

**Authors:** N. A. Borghese, G. Bottini, A. Sedda

**Affiliations:** ^1^Laboratory of Applied Intelligent Systems, Computer Science Department, University of MilanMilan, Italy; ^2^Department of Psychology, University of PaviaPavia, Italy; ^3^Cognitive Neuropsychology Center, Niguarda Ca’ Granda HospitalMilan, Italy

Post-stroke recovery is negatively affected by the presence of visuo-spatial neglect: patients with this diagnosis are more impaired in terms of independence, get lower scores on disability tests, and require longer rehabilitation period (Stone et al., 1992; Katz et al., 1999; Di Monaco et al., 2011). In light of the functional implications that characterize this pathology, it is not surprising that the development of efficient rehabilitation techniques is an important aim of the present research on neglect (Cappa, 2008). Videogames (VG) may offer an effective alternative to traditional behavioral and cognitive rehabilitation as they can integrate cognitive training with high flexibility in a daily life scenario (Rose et al., 2005; Tsirlin et al., 2009). The introduction of low cost, effective tracking devices like Sony's PlayStation Eye™ and PlayStation Move™, Nintendo's Wii Remote Plus™, and Microsoft's Kinect™ were soon recognized as a major source of inspiration for rehabilitation. However, commercially available VG, developed with the aim of amusement, do not match rehabilitation guidelines (i.e., use of meaningful functional activities, management of cognitive impairments through compensatory strategies and retraining skills (Wilson, 2008) posing the question of their applicability in this domain (Laver et al., 2011a). For this reason, *ad hoc* VG engines have been developed, based on these tracking devices, that do provide the monitoring and adaptation capabilities required by rehabilitation games (Pirovano et al., 2012). With a careful design of the virtual environments, rehabilitation sessions can become even more engaging for patients and increase their motivation (Thornton et al., 2005; Laver et al., 2011b; Pirovano et al., 2012). Enriched environments (Risedal et al., 2002), intense practice (Nudo et al., 2001), the possibility to tailor a rehabilitation session to patient's needs, to tune the degree of difficulty to patient's competences, and to enhance interaction during rehabilitation through an immediate feedback to the patient (Sveistrup, 2004) are the most promising features of VG approaches. It has also been suggested that a scenario including meaningful objects, rather than abstract geometric targets as stimuli, could be more motivating and encouraging for patients engaged in motor recovery programs leading to positive outcomes (Laver et al., 2011b; Sedda et al., 2013; Mainetti et al., 2013).

Nowadays, however, a simple view based only on the features of the programs cannot explain results obtained through VG platforms. Possible underlying mechanisms of brain reorganization after rehabilitation in virtual environments are unclear and could be far more complex. Nevertheless, there are several clues that they could ground mainly on two processes: (i) near/far spatial remapping, and (ii) multisensory integration. The role of these processes in recovery may be due to the multi-componential character of neglect syndrome (Milner and McIntosh, 2005; Hillis, 2006), as it may affect various domains, such as perception and mental representation in multiple sensory modalities.

Remapping of space (Berti and Frassinetti, 2000; Berti et al., 2001; Ansuini et al., 2006) is strongly connected to updating of the body schema representation (Neppi-Modona et al., 2007; Sedda et al., 2013; Mainetti et al., 2013), and may involve an action component which is associated with dorsal stream processing (Neppi Modona et al., 2007; Sedda and Scarpina, 2012). The concepts of near/far space and reaching/locomotion can be can be taken into account as good examples to understand why more exhaustive models that considers the above mentioned concepts are needed. Reaching is an action that allows to bring the hand near to an object or to a spatial location. Consequently, space can be divided into within-reachable distance (near) and beyond-reachable distance (far). One peculiar feature of VG treatments is that although trained only in the far space, patients recover from neglect also in the peripersonal space (Kim et al., 2011; Sedda et al., 2013; Mainetti et al., 2013). In the past, the idea that an action could boost remapping of near-far space has been explored also with regards to locomotion (Berti et al., 2002), an action more often performed in everyday life than the grasping with tools. Locomotion involves the use of legs and allows humans and animals to move in space and change their position. The logic beyond this tentative experiment was mainly grounded on two assumptions: (i) far space is coded based on retinal coordinates, while near space is coded based on egocentric coordinates [meaning that in one case spatial position is reconstructed by computing the position of an object on the retina and the position of the eye in the orbit, while in the other case this computation is related not only to the body midline but also to body parts (Berti et al., 2002)] and (ii) locomotion is an effective action to reach the space in which a target object to be grasped is placed (Berti et al., 2002). However, this research highlighted that, at least for short, linear trajectories, remapping of space does not occur in neglect patients. A possible explanation for the failure of spatial remapping during walking is that locomotion is only a mean to reach a location, but the action plans related to grasping are not active yet. In fact locomotion has its own neural networks (Sahyoun et al., 2004), makes use of different effectors than hand movements, and one can assume that during locomotion only the generic distance between the body and the object is computed, while fine graded movements representation are activated later, only when the hand is approaching the object. Furthermore, action representation for walking and for grasping are quite diverse and do not completely overlap (Sahyoun et al., 2004). The difference between these actions explains why models need to take into account also the concept of dorsal stream. The dorsal stream is devoted to planning and control of actions such as reaching and grasping, that require coordination between fingers, hands, and eyes as well as the computation of object size, their distance from the hand, their position in terms of egocentric coordinates, and in relation to a dynamic world in which targets and obstacles are moving (Sedda and Scarpina, 2012). Not all these features are considered in locomotion planning: for instance, object's size is not processed when planning to walk. Consequently, to parallel tools use to walking (Berti et al., 2002) one should assume that locomotion representations should transfer to hand grasping representations for a remapping to take place. Differently, when grasping with a tool and grasping with the hand, functionally related body segments are involved, allowing possibly an easier transferability of activations. Furthermore, the same features of the object are processed. Specifically, one can hypothesize that in case of a grasping movement the spatial remapping occurs due to the “action feedback” in the absence of a tactile or visual continuity obtained by means of a tool allowing to reach the far space (i.e., a long stick or a laser pointer) (Neppi-Modona et al., 2007). Congruently, recent studies suggest that the active visuo-motor learning of using a tool rather than its passive holding, leads to spatial adaptation and influences representation of space (Brown et al., 2011). This result strongly suggest an involvement of the dorsal stream in spatial remapping, at least when hand actions are required. This explanation might partially account for the success of VG based techniques making use of far (virtual) space to rehabilitate neglect also in peripersonal space. Performing real and functionally meaning actions could boost a spatial remapping more than a button press or walking toward the target. These hypotheses suggest that actions to be employed in VG base treatments should be carefully chosen.

In such view, however, it is necessary to consider that the well-known dissociation far and near space is not exhaustive to explain treatment success. One may speculate further that the multisensory integration facilitated by the immediate feedback provided by seeing one owns upper limb reflected in the far space while reaching objects, might facilitate the spatial remapping between far and near space through the updating of the body schema, which is strongly dependent on multisensory integration (Aglioti et al., 1996; Iriki et al., 1996; Berti and Frassinetti, 2000; Farne and Ladavas, 2000; Neppi-Modona et al., 2007; Sedda and Scarpina, 2012). Body schema refers to a dynamic representation of body parts in space, continuously updated during movement, distinct from the conscious and semantic description of the body that we can reach through awareness (Berlucchi and Aglioti, 2010). Implicit in this definition of body schema is its strong link with actions such as grasping and reaching. Importantly, VG treatments are more and more making use of the real dynamic silhouette of patients (Kim et al., 2011; Sedda et al., 2013; Mainetti et al., 2013). Rehabilitation platforms providing patients with their own image (Kim et al., 2011; Sedda et al., 2013; Mainetti et al., 2013) instead of avatars might favor the re-adaptation of a compromised body schema in an easier way than through cognition, as humans see their mirrored body since childhood (Beis et al., 2001). The patient is able to see his upper limb reflected into the virtual environment allowing him to perceive his movements time by time, benefiting unconsciously from the spatio-temporal congruency between real and virtual arm. Moreover the use of mirror images seems to improve the performance of right brain damaged patients with neglect when reaching objects located in the contralesional, ignored space (Ramachandran et al., 1999). In patients without cognitive dysfunctions such as spatial recalibration deficits or mirror ataxia (Beis et al., 2001) the real silhouette method might be a powerful mean to activate dorsal stream circuits, allowing a more fruitful rehabilitation path.

As a final remark, effectiveness of VG based treatments on diverse subtypes of neglect should be explored. For instance, these techniques might not be suitable for all neglect patients, considering that additional impairments such as somatoparaphrenia or perseverations might be present (Bottini et al., 2009). Somatoparaphrenia impacts body representation, while perseverations make visual search far more difficult. Together, somatoparaphrenia and perseverations undermine the interactive component of VG based tasks. Further, effectiveness of VG based treatment of patients with near or far only neglect might be different (Halligan and Marshall, 1991; Vuilleumier et al., 1998; Keller et al., 2005; Aimola et al., 2012) and should be explored. One could question whether patients showing only far neglect, not having near neglect, would not show the observed remapping between far and near space. It is not known whether these patients would improve in far space, as available studies only investigated the outcome in near space (Kim et al., 2011; Sedda et al., 2013; Mainetti et al., 2013). This implies that VG inspired studies should also adopt more fine graded assessment of neglect and related impairments, and samples selected *ad hoc* to allow within group contrasts, aimed at verifying the suitability of paradigms across different neglect subtypes.

Appropriate rehabilitation techniques may influence cognitive functions even in the chronic phase (Teasell et al., 2005). A *wider and enriched scenario* including *meaningful actions*, rather than abstract geometric targets as stimuli and movements that do not resemble reality, is more motivating, encouraging, and finally ecological for patients engaged in recovery programs (Laver et al., 2011b). For neglect patients, revisiting the classical visual search tasks (Bowen and Lincoln, 2007) through a VR environment might ensure more effective results not only because of the technological advanced equipment, but because this equipment allows to transfer classical theoretical concepts (such as those of body schema and action planning) in the rehabilitation field. New paradigms programing should take into account these theories and should try to integrate as many as possible of their principles, to reach optimal results in terms of impact on patients recovery.
